# Chronic Treatment with Anti-bipolar Drugs Down-Regulates Gene Expression of TRPC1 in Neurones

**DOI:** 10.3389/fncel.2016.00305

**Published:** 2017-01-10

**Authors:** Ting Du, Yan Rong, Rui Feng, Alexei Verkhratsky, Liang Peng

**Affiliations:** ^1^Laboratory of Metabolic Brain Diseases, Institute of Metabolic Disease Research and Drug Development, China Medical UniversityShenyang, China; ^2^Faculty of Life Sciences, The University of ManchesterManchester, UK; ^3^Achucarro Center for Neuroscience, Basque Foundation for ScienceBilbao, Spain

**Keywords:** neurone, bipolar disorder, carbamazepine, lithium salts, valproic acid, TRPC1 channels, SOCE

## Abstract

In the brain, TRPC1 channels are abundantly expressed in neurones virtually in all regions; these proteins function as receptor-activated ion channels and are implicated in numerous processes, being specifically important for neurogenesis. Primary cultures of mouse cerebellar granule cell, cerebral cortical neurones, and freshly isolated neurones from *in vivo* brains were used to study effects of chronic treatment with anti-bipolar drugs [carbamazepine (CBZ), lithium salts and valproic acid] on gene expression of TRPC1. Expression of TRPC1 mRNA was identified with reverse transcription-polymerase chain reaction, whereas protein content was determined by Western blotting. Store-operated plasmalemmal Ca^2+^ entry (SOCE) was measured with fura-2 based microfluorimetry. Chronic treatment with each of the three drugs down-regulated mRNA and protein expression in cultured cerebellar granule cells in a time- and concentration-dependent manner. Similar effect was also observed in cultured cerebral cortical neurones treated with CBZ, lithium salts and valproic acid and in freshly isolated neurones from the brains of CBZ-treated animals. The amplitude of SOCE was substantially decreased in cerebellar granule cells chronically treated with each of the three drugs. Our findings indicate that down-regulation of TRPC1 gene expression and function in neurones may be one of the mechanisms of anti-bipolar drugs action.

## Introduction

Bipolar disorder (BD) is a common, devastating and chronic mental disease that affects 1–3% of the population (Goodwin and Jamison, 2007). Carbamazepine (CBZ), lithium salts (Li^+^), and valproic acid (VPA) are the three classical anti-bipolar drugs. However, despite being in clinical use for several decades, the mechanisms underlying their therapeutic effects remain elusive. Given CBZ, Li^+^, and VPA share no similarity in chemical structures, revealing downstream effects of all three drugs is important for further understanding drug targets and the pathophysiology of the disease. We have found several genes that are regulated by chronic treatment with anti-bipolar drugs in astrocytes (for review, see Peng et al., 2016). These includes down-regulation of gene expression of glutamate kainate receptor GluK2 (Li et al., 2009), the Ca^2+^-dependent phospholipase A_2_ (Li et al., 2007) and transient receptor potential channel 1 (TRPC1); a cation channel, which in astroglia is activated by ER store depletion and contributes to the store-operated Ca^2+^ entry, SOCE (Yan et al., 2013).

TRPC1 channels are abundantly expressed in all regions of the brain, including cortex, hippocampus, cerebellum, forebrain, brain stem and basal ganglia (Riccio et al., 2002; Martorana et al., 2006; Narayanan et al., 2008 and see Vennekens et al., 2012 for comprehensive review). In the early neurogenesis TRPC1 channels have been proven to be critical, because they control proliferation of adult neural progenitor cell, and genetic deletion of TRPC1 significantly reduced proliferation rate of these progenitors. Importantly the contribution of TRPC1 to regulation of proliferation was directly linked to TRPC1-mediated SOCE (Li et al., 2012). In addition TRPC1 (together with TRPC5 and TRPC6) regulate neurite extension, most likely through fine tuning Ca^2+^ signals in the growth cone (Vennekens et al., 2012).

We are therefore, interested in whether canonical anti-bipolar drugs have effect on TRPC1 gene expression in neurones. In the present paper, we report experiments on primary cultures of mouse cerebellar granule neurones, cerebral cortical neurones, and freshly isolated neurones from transgenic mice expressing a fluorescent neurone-specific marker used for fluorescence-activated sorting (FACS). Cerebellar granule cells represent a well-established, well-differentiated model for studying glutamatergic signaling (Gallo et al., 1987; Marini et al., 1989; Peng et al., 1991; Bak et al., 2003); granule neurones express NMDA, AMPA and metabotropic glutamate receptors, and they are vulnerable to excitotoxic insults (Condorelli et al., 1993; Lavreysen et al., 2005). Cerebral cortical neurones are GABAergic and also sensitive to glutamate excitotoxicity mediated by NMDA receptors (Chuang et al., 2002), whereas in experiments *in vivo* analysis was mainly restricted to large projection neurones which express Thy1 promoter. We studied: (i) effects of chronic treatment with anti-bipolar drugs on mRNA and protein expression of TRPC1 in primary cultures of cerebellar granule neurones; (ii) effects of chronic treatment with anti-bipolar drugs on mRNA and protein expression of TRPC1 in primary cultures of cerebral cortical neurones; (iii) expression of TRPC1 mRNA in FACS-isolated neurones from animals treated with daily injections of CBZ, and (iv) effects of chronic treatment with anti-bipolar drugs on SOCE in primary cultures of cerebellar granule cells.

## Materials and Methods

### Animals

Male B6.Cg-Tg(Thy1-YFPH)2Jrs/J mice (from The Jackson Laboratory, Bar Harbor, ME, USA), weighing 20–25 g were housed in cages on a 12 h light/dark cycle in a temperature-controlled (23–25°C) colony room with free access to food and water. These transgenic mice express fluorescent neurone-specific marker, allowing fluorescence-activated sorting of specified cell fractions, although it should be emphasized that Thy1 is mainly a marker of large projection neurones rather than a general neuronal marker (Feng et al., 2000; Seki et al., 2002). All experiments were carried out in accordance with the USA National Institute of Health Guide for the Care and Use of Laboratory Animals (NIH Publications No. 80-23) revised 1978, and all experimental protocols were approved by the Institutional Animal Care and Use Committee of China Medical University.

### Cell Cultures

Cerebellar granule cells were cultured as previously described (Peng et al., 1991) with minor modifications. Briefly, 7- day-old mouse pups were rapidly decapitated and the brains taken out. The cerebella were aseptically separated from the remainder of the brain, and after removal of the meninges, the tissue was cut into cubes of -0.4 mm side dimensions, exposed to trypsin in a Ca^2+^/Mg^2+^-free salt solution, reintroduced into tissue culture medium, passed through nylon sieves and seeded into polylysine coated standard 35-mm tissue culture dishes (Wuzhou Medical Plastic Factory, Zhejiang, China), using one cerebellum per culture dish. The cultures were grown in Dulbecco’s medium in which the glucose concentration was increased to 30 mM and the K^+^ concentration to 24.5 mM, the glutamine concentration was decreased to 0.8 mM and 7% horse serum was added. The elevation of the K^+^ concentration is necessary for normal development of the cells (Gallo et al., 1987) and more specifically for normal dendritic development and for release of transmitter glutamate (Peng et al., 1991). After 2 days, cytosine arabinoside was added to the medium to a final concentration of 40 μM to curtail astrocytes growth. The cells were used at the age of 7–8 days at which time they have reached maturity (Peng et al., 1991).

Cerebral cortical neurones were prepared by a similar method as the cerebellar granule neurones (Hertz et al., 1989) except that cortical hemispheres from 14-day-old mouse embryos were used as the source of the cultures and that the cultures were grown in normal tissue culture medium with 5.4 mM K^+^ and 2 mM glutamine. The cultures were used at 8–10 days of age when they have developed characteristics of mature cortical interneurones (Schousboe and Hertz, 1987).

### Drug Treatment

Adult mice were daily injected intraperitoneally with CBZ [25 mg/kg/d dissolved in 0.9% NaCl (Rao et al., 2007)] or saline for 3 or 7 days. Three days after plating primary cultures of cerebellar granule cells and cerebral cortical neurones were treated with CBZ, lithium carbonate or VPA for either 3 or 7 days.

### Preparation of Freshly Isolated Cells

Immediately after decapitation, cerebral hemispheres (without olfactory bulbs and hippocampi) were removed. The remaining parts of the brains were placed in cold Hanks buffer containing glutamate receptor antagonists (3 μM DNQX and 100 μM APV), cut into small pieces, and digested with 8 U/ml papain in Ca^2+^/Mg^2+^-free PIPES/cysteine buffer, pH 7.4, for 1 h at 37°C in a humidified atmosphere of CO_2_/air (5:95%). After one wash, the tissue was further digested with 40 U/ml DNase I in Mg^2+^-containing minimum essential medium (MEM) with 1% bovine serum albumin (BSA) for 15 min at 37°C in a humidified atmosphere of CO_2_/air (5:95%). It was then carefully triturated in cold MEM with 1% BSA, and centrifuged over a 90% Percoll gradient, followed by collection of all cells below and including the lipid layer. This suspension was further diluted five times, with MEM containing 1% BSA, and centrifuged to collect the pellet. Immediately thereafter, the cells were re-suspended in cold MEM with 1% BSA and 4 μg/ml propidium iodide and sorted by FACS using the BD FACSAria Cell Sorting System (35 psi sheath pressure, FACSDiva software S/W 2.2.1; BD Biosciences, San José, CA, USA) as described by Lovatt et al. (2007). YFP were excited by a 488 nm laser, and emissions were collected by 530 nm discrimination filters. Since a relatively small amount of cells was obtained following FACS-separation we performed only mRNA detection. Cell identity and purity were verified by mRNA expression of cell markers of astrocytes, neurones and oligodendrocytes, analyzed by reverse-transcription polymerase chain reaction (RT-PCR), in astrocytic and neuronal cell preparations. As shown by Fu et al., 2012 there is no contamination with neuronal or oligodendrocytic genes in the samples of astrocytes or of astrocytic or oligodendrocytic genes in the neuronal samples.

### Reverse Transcription-Polymerase Chain Reaction (RT-PCR)

For determination of mRNA expression by RT-PCR, a cell suspension was prepared by discarding the culture medium, adding Trizol to cultures on ice, and scraping the cells off the culture dish. The RNA pellet was precipitated with isopropanol, washed with 70% ethanol, and dissolved in 10 μl sterile, distilled water, and an aliquot was used for determination of the amount of RNA (Kong et al., 2002).

Reverse-transcription was initiated by a 5 min-incubation at 65°C of 1 μg RNA extract with Random Hexamer at a final concentration of 12.5 ng/l and deoxy-ribonucleoside triphosphates (dNTPs) at a final concentration of 0.5 mM. The mixture was rapidly chilled on ice and briefly spun, and 4 μl 5× First-Strand Buffer, 2 μl 0.1 M dithiotreitol and 1 μl RNaseOUT Recombinant RNase Inhibitor (40 U/μl) were added. After the mixture had been incubated at 42°C for 2 min, 1 μl (200 U) of Superscript II was added, and the incubation at 42°C continued for another 50 min. Subsequently, the reaction was inactivated by heating to 70°C for 15 min, and the mixture was chilled and briefly centrifuged.

Polymerase chain reaction (PCR) amplification was performed in a Robocycler thermocycler with 0.2 μM of sense or antisense and 0.375 U of Taq polymerase for TRPC1 (forward, CCTTCTCATACTGTGGATTATTG; reverse, GTACCAGAACAGAGCAAAGCA) (Ng et al., 2009), and for TATA box-binding protein (TBP), used as a housekeeping gene (forward, CCACGGACAACTGCGTTGAT; reverse, GGCTCATAGCTACTGAACTG) (el-Marjou et al., 2000), designed and purchased form Sangon Company (Shanghai, China). Initially the template was denatured by heating to 94°C for 2 min, followed by 2.5 min amplification cycles, each consisting of two 45-s periods and one 60-s period, the first at 94°C, the second at 58°C for TRPC1 and at 55°C for TBP, and the third at 72°C. The final step was extension at 72°C for 10 min. The PCR products were separated by 1% agarose gel electrophoresis, stained with 0.5 μg/ml ethidium bromide, and captured by Fluorchem 5500 (Alpha Innotech Corporation, San Leandro, CA, USA).

### Western Blotting

Protein content was determined by the Lowry method (Lowry et al., 1951), using BSA as the standard. Samples containing 100 μg protein for TRPC1 or 20 μg for β-actin were applied on slab gels of 10% polyacrylamide and electrophoresed. After transfer to polyvinylidene fluoride (PVDF) membranes, the samples were blocked by 5% skim milk powder in TBS-T (30 mM Tris-HCl, 125 mM NaCl, 0.1% Tween 20) for 1 h. The PVDF membranes were incubated with the first antibody, specific to TRPC1 overnight at 4°C, or β-actin for 2 h at room temperature. After washing, the blots were incubated with peroxidase-conjugated affinity-purified goat-anti-mouse or goat-anti-rabbit horseradish antibody for 2 h. Staining was visualized by ECL detection reagents. Digital images obtained using Gel-Imaging System (Tanon, 4200, Shanghai). Optical density for each band was assessed using the Window Alpha-Ease TM FC 32-bit software.

### Monitoring of Intracellular Ca^2+^ Concentration

For [Ca^2+^]_i_ recordings an Olympus IX71 live cell imaging fluorescence microscope (Tokyo, Japan) was used to monitor fluorescence intensity of Fura-2 loaded cultured neurones. For Fura-2/AM loading the growth medium was replaced with saline solution (137 mM NaCl, 5 mM KCl, 0.44 mM KH_2_PO_4_, 4 mM NaHCO_3_, 1.3 mM CaCl_2_, 0.8 mM MgSO_4_, and 0.5 mM MgCl_2_ with 10 mM glucose) containing 5 μM Fura-2/AM for 30 min at 37°C. After two times wash with similar saline, the coverslip was perfused with the saline solution, and recordings were made at 340 and 380 nm excitation and 510 nm emission wavelengths at 15 s intervals. The data are presented as 340/380 ratio R normalized to 340/380 ratio reading at the rest (R_0_). The SOCE was assessed by measuring the amplitude of [Ca^2+^]_i_ transient induced by re-addition of extracellular Ca^2+^ to cells pre-incubated with Ca^2+^-free buffer in combination with the SERCA inhibitor thapsigargin. Twenty cells were selected in each coverslip, and three coverslips were averaged in each experimental group.

### Materials

Chemicals for preparation of culturing medium were purchased from Sigma (St. Louis, MO, USA) and horse serum from Invitrogen (Carlsbad, CA, USA). Most chemicals, including CBZ, VPA, 4-CML (4-chloro-3-methylphenol) and thapsigargin were purchased from Sigma (St. Louis, MO, USA). Lithium carbonate (Li_2_CO_3_) was obtained from Shanghai Hengxin Chemical Reagent (Shanghai, China). TRPC1 antibody (ACC-010) was obtained from Alomone Labs (Jerusalem, Israel). Antibody specific to β-actin (A5441) was obtained from Sigma-Aldrich (St. Louis, MO, USA). Goat-anti-rabbit IgG HRP conjugate was obtained from Santa Cruz Biotechnology (Santa Cruz, CA, USA). Goat-anti-mouse IgG HRP conjugate was obtained from Promega (Madison, WI, USA). Fura-2 AM was obtained from Invitrogen (Carlsbad, CA, USA). Random Hexamer and Taq-polymerase for RT-PCR were purchased from TaKaRa Biotechnology, Co., Ltd (Dalian, China).

### Statistics

All statistical comparisons between groups were performed by Student’s *t*-test. Levels of significance were expressed as *P*-values.

## Results

### Gene Expression of TRPC1

**FIGURE 1:**
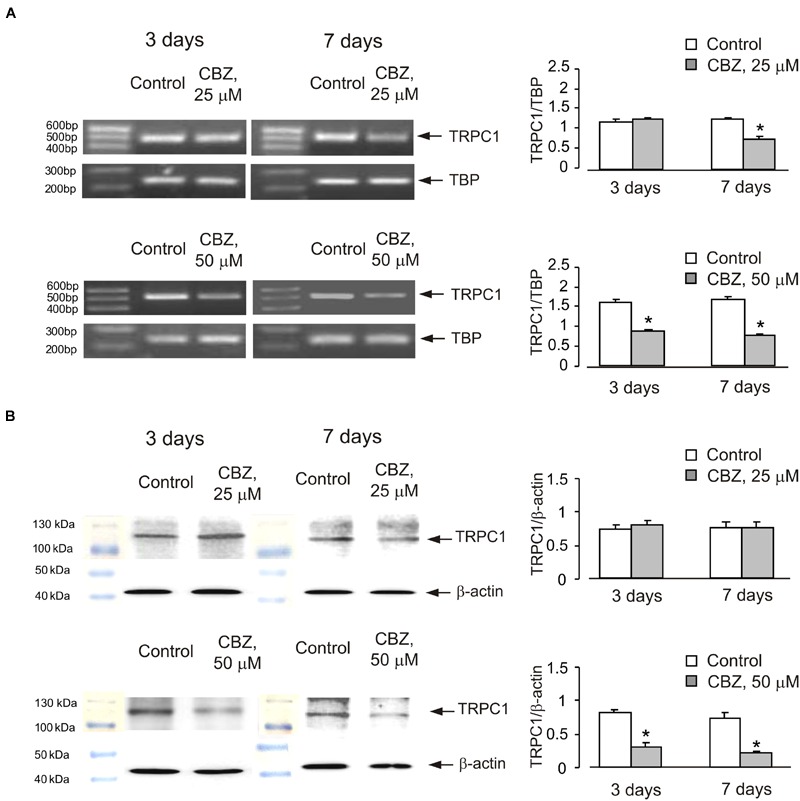
**Down-regulation of mRNA and protein expression of TRPC1 by chronic treatment with carbamazepine (CBZ) in primary cultures of mouse cerebellar granule cells.** Cells were treated with PBS (control) or with 25 or 50 μM CBZ for 3 or for 7 days. **(A)** Southern blot from a representative experiment. Similar results were obtained from three independent experiments. Average mRNA expression was quantified as a ratio between TRPC1 and TBP, as a housekeeping gene. The size of the PCR product of TRPC1 is 517 bp and that of TBP 236 bp. SEM values are indicated by vertical bars. ^∗^Statistically significant (*p* < 0.05) difference from control group. **(B)** Immunoblot from a representative experiments. Similar results were obtained from three independent experiments. Average protein expression was quantified as a ratio between TRPC1 and β-actin. SEM values are indicated by vertical bars. ^∗^Statistically significant (*p* < 0.05) difference from control group.

Chronic treatment of cultured cerebellar granule neurones with CBZ decreased mRNA and protein expression of TRPC1 in a time- and concentration-dependent manner (**Figure 1**). Seven days of treatment with CBZ at 25 μM down-regulated of mRNA expression of TRPC1 whereas 3 days of treatment had no effect. However, CBZ at 50 μM caused a significant down-regulation of TRPC1 mRNA after 3 days (54 ± 4.1%) with further decline after 7 days [45 ± 4.6% of control group (**Figure 1A**)]. Protein expression of TRPC1 was unaffected by both 3 and 7 days of treatment with 25 μM CBZ, but after 3 or 7 days of treatment with 50 μM CBZ it decreased significantly to 37 ± 9.1 and 30 ± 3.9%, respectively when compared to the control group (**Figure 1B**; originals of blots are presented in **Supplementary Figure S1**).

**FIGURE 2:**
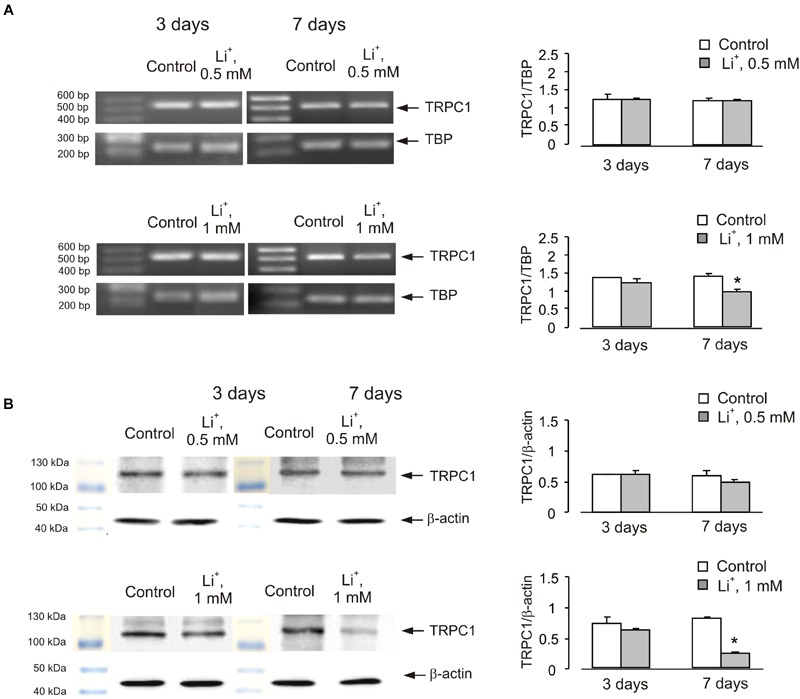
**Down-regulation of mRNA and protein expression of TRPC1 by chronic treatment with lithium in primary cultures of mouse cerebellar granule cells.** Cells were treated with PBS (control) or with 0.5 or 1 mM lithium for 3 or 7 days. **(A)** Southern blot from a representative experiment. Similar results were obtained from three independent experiments. Average mRNA expression was quantified as a ratio between TRPC1 and TBP, as a housekeeping gene. The size of the PCR product of TRPC1 is 517 bp and that of TBP 236 bp. SEM values are indicated by vertical bars. ^∗^Statistically significant (*p* < 0.05) difference from control group. **(B)** Immunoblot from a representative experiments. Similar results were obtained from three independent experiments. Average protein expression was quantified as a ratio between TRPC1 and β-actin. SEM values are indicated by vertical bars. ^∗^Statistically significant (*p* < 0.05) difference from control group.

**FIGURE 3:**
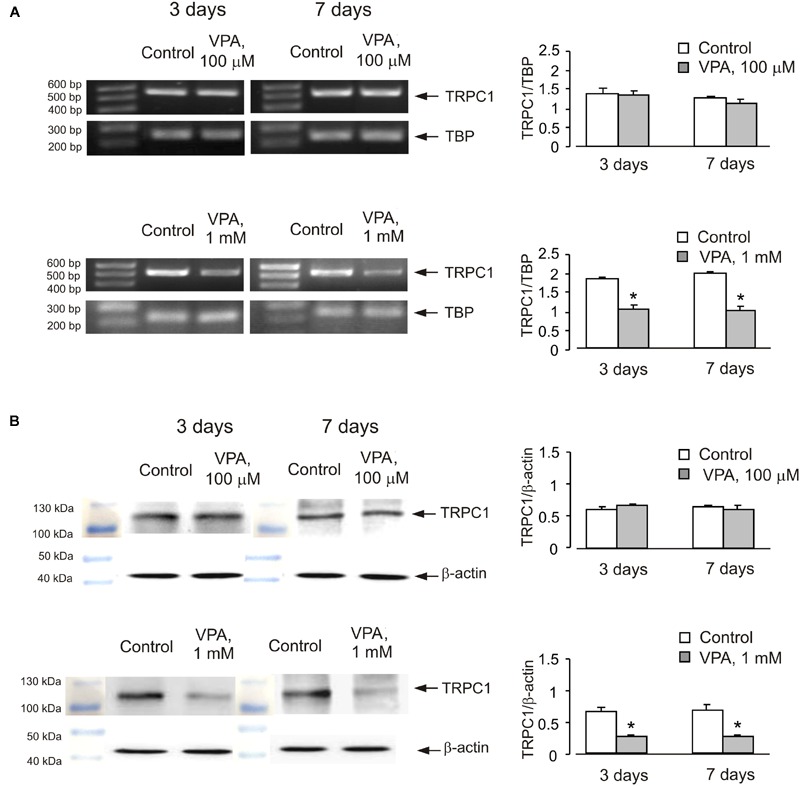
**Down-regulation of mRNA and protein expression of TRPC1 by chronic treatment with VPA in primary cultures of mouse cerebellar granule cells.** Cells were treated with PBS (control) or with 100 μM or 1 mM VPA for 3 or 7 days. **(A)** Southern blot from a representative experiment. Similar results were obtained from three independent experiments. Average mRNA expression was quantified as a ratio between TRPC1 and TBP, as a housekeeping gene. The size of the PCR product of TRPC1 is 517 bp and that of TBP 236 bp. SEM values are indicated by vertical bars. ^∗^Statistically significant (*p* < 0.05) difference from control group. **(B)** Immunoblot from a representative experiments. Similar results were obtained from three independent experiments. Average protein expression was quantified as a ratio between TRPC1 and β-actin. SEM values are indicated by vertical bars. ^∗^Statistically significant (*p* < 0.05) difference from control group.

Similar effect was also observed in cells treated with lithium carbonate or VPA (**Figures 2** and **3**). Exposure to 0.5 mM Li^+^ did not affect TRPC1 expression neither after 3 nor after 7 days, whereas a decrease in expression to 61 ± 3.4% of control group was observed at 1 mM Li^+^ after 7 days (**Figure 2A**). Protein expression of TRPC1 has not changed after 3 as well as after 7 days of treatment with 0.5 mM Li^+^. However, after 7 days of treatment with 1 mM Li^+^, protein expression of TRPC1 significantly decreased to 31 ± 6.9% compared to control (**Figure 2B**). Similarly, exposure to 100 μM VPA had no effect, whereas in the presence of 1 mM VPA, mRNA expression of TRPC1 decreased to 55 ± 5.5 and 51 ± 5.8% of control group after 3 and 7 days, respectively (**Figure 3A**). The TRPC1 protein was similarly down-regulated in the presence of 1 mM VPA after 3 days to 40 ± 8.3% of control group and after 7 days to 39 ± 4.1% of control group, although no effect was detected at 100 μM of VPA after 3 and 7 days (**Figure 3B**).

**FIGURE 4:**
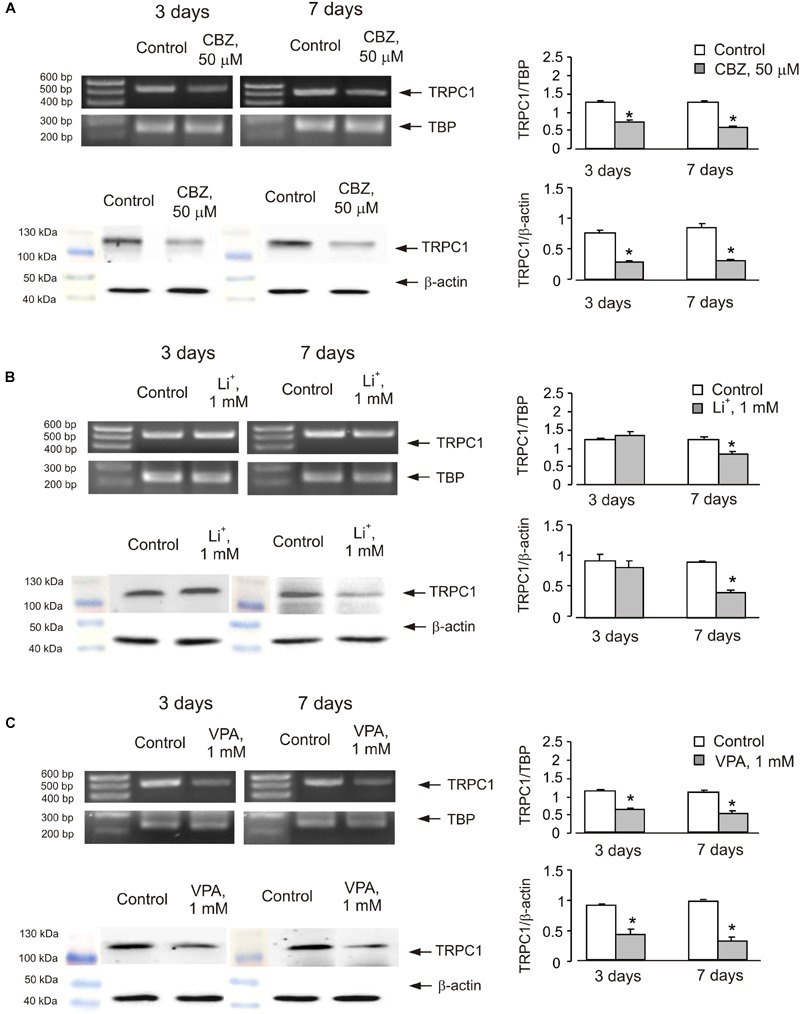
**Down-regulation of mRNA and protein expression of TRPC1 by chronic treatment with CBZ, Li^+^, or VPA in primary cultures of mouse cerebral cortical neuron. (A)** Cells were treated with PBS (control) or with 50 μM CBZ for 3 or 7 days. **(B)** Cells were treated with PBS (control) or with1 mM Li^+^ for 3 or 7 days. **(C)** Cells were treated with PBS (control) or with 1 mM VPA for 3 or 7 days. The representative Southern blot and immunoblot are shown. Similar results were obtained from three independent experiments. Average mRNA expression was quantified as a ratio between TRPC1 and TBP, as a housekeeping gene. The size of the PCR product of TRPC1 is 517 bp and that of TBP 236 bp. Average protein expression was quantified as ratios between TRPC1 and β-actin. SEM values are indicated by vertical bars. ^∗^Statistically significant (*p* < 0.05) difference from control group.

**FIGURE 5:**
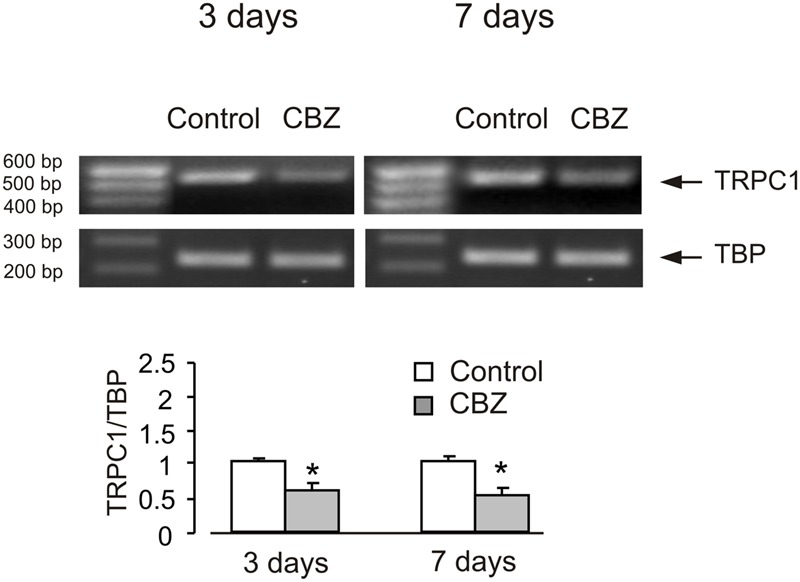
**CBZ induced down-regulation of mRNA and protein expression of TRPC1 in neurones isolated by FACS from cerebral hemisphere *in vivo* from mice.** Adult [B6.Cg-Tg(Thy1–YFPH)2Jrs/J] mice were treated with either saline (control) or CBZ (25 mg/kg/day) dissolved in saline for 3 or 7 days. Average mRNA expression was quantified as a ratio between TRPC1 and TBP, as a housekeeping gene. The size of the PCR product of TRPC1 is 517 bp and that of TBP 236 bp. Similar results were obtained from three independent experiments. SEM values are indicated by vertical bars. ^∗^Statistically significant (*p* < 0.05) difference from control group.

In order to see whether the effect of anti-bipolar drugs is restricted to cerebellar granule cells, we analyzed the effect of CBZ, lithium carbonate or VPA on TRPC1 gene expression in cultured cerebral cortical neurones. Treatment of cultured cerebral cortical neurones with 50 μM CBZ induced a decrease of TRPC1 mRNA expression to 57 ± 2.1 and 47 ± 2.0% of control group after 3 and 7 days, respectively. Protein expression of TRPC1 similarly decreased to 39 ± 1.8 and 35 ± 2.5% compared to control after 3 and 7 days of treatment (**Figure 4A**). Exposure to 1 mM lithium carbonate decreased TRPC1 mRNA expression to 68 ± 2.1% of control group after 7 days. Protein expression of TRPC1 similarly decreased to 47 ± 3.5% compared to control after 7 days of treatment (**Figure 4B**). Treatment with 1 mM VPA decreased TRPC1 mRNA expression to 51 ± 1.6 and 49 ± 2.2% of control group after 3 and 7 days, respectively. Protein expression of TRPC1 was similarly decreased to 47 ± 7.6 and 32 ± 5.8% compared to control after 3 and 7 days of treatment (**Figure 4C**). In addition, comparable decrease in TRPC1 mRNA expression was observed in neurones freshly isolated from the brains of CBZ treated mice; mRNA was decreased to 61 ± 5.2 and 52 ± 2.0% of control group after 3 and 7 days, respectively (**Figure 5**).

### Store-Operated Ca^2+^ Entry

**FIGURE 6:**
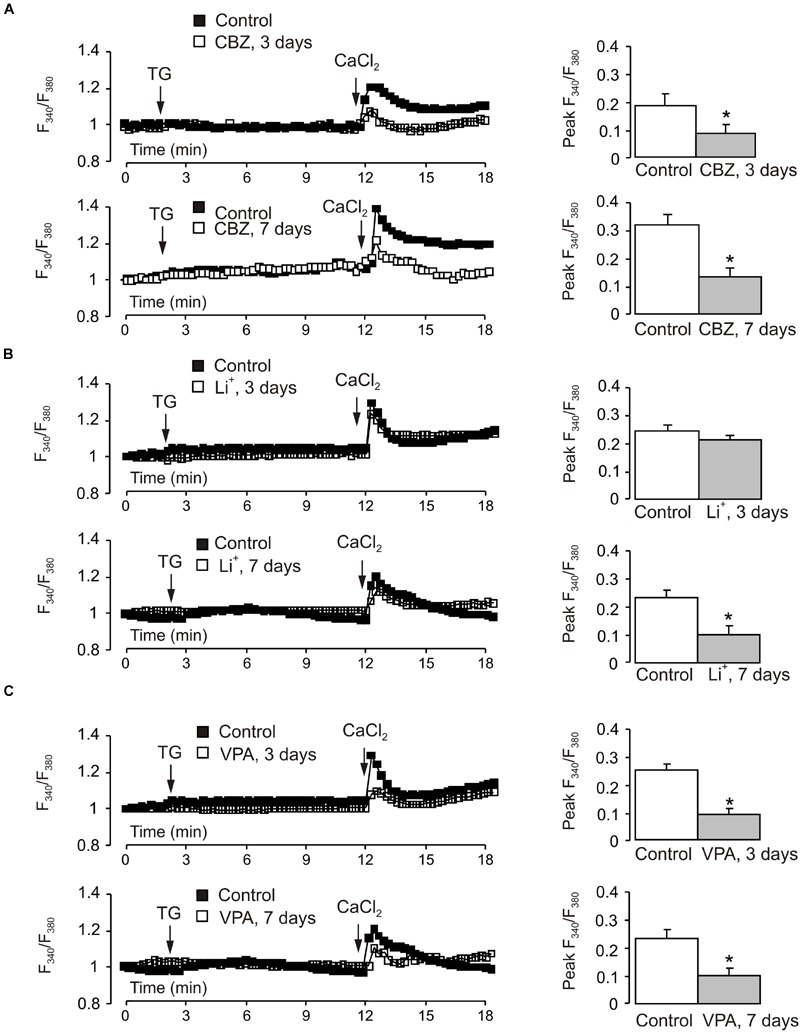
**Effect of chronic treatment with CBZ, lithium or VPA on store-operated Ca^2+^ entry in primary cultures of mouse cerebellar granule cells.** Cells were treated with CBZ (50 μM), lithium (1 mM), or VPA (1 mM) for 3 or 7 days. After loading with fura-2 AM for 30 min, cells were incubated in Ca^2+^-free buffer with 1 μM thapsigargin, an inhibitor of SERCAs. After 10 min Ca^2+^ (2 mM) was re-introduced into the incubation buffer. **(A–C)** Representative traces show the averaged changes in [Ca^2+^]_i_ (normalized with the R/R_0_ ratio at the 0 time; each representative trace shows the average changes in [Ca^2+^]_i_ of three individual experiments; 20 cells per experiment). Mean of peak amplitudes of SOCE-associated [Ca^2+^]_i_ peak. SEM values are indicated by vertical bars. ^∗^Shows statistically significant (*p* < 0.05) difference from control group.

The decrease of TRPC1 gene expression in cerebellar granule cells chronically treated with CBZ was associated with the decrease in the amplitude of SOCE (**Figure 6A**). To analyze the SOCE cells were incubated in Ca^2+^-free buffer with 1 μM thapsigargin for 10 min. Thereafter Ca^2+^ (2 mM) was re-introduced into the incubation buffer, this triggering [Ca^2+^]_i_ transient mediated by the SOCE pathway. Of note, the SOCE was not affected by inhibitors of voltage-gated Ca^2+^ channels (**Supplementary Figure S2**), indicating that SOCE-associated cell depolarisation did not exceed the threshold of Ca^2+^ channels activation. The amplitude of [Ca^2+^]_i_ transient associated with SOCE decreased by ∼55% (0.1894 ± 0.0290 in control vs. 0.0857 ± 0.0228 in CBZ treated cells, *n* = 60, *p* < 0.05) after 3 days and by ∼58% (0.3217 ± 0.0394 in control vs. 0.1361 ± 0.0313 in CBZ treated cells, *n* = 60, *p* < 0.05) after 7 days of treatment with 50 μM CBZ (**Figure 6A**). Treatment with 1 mM Li^+^ for 3 days had no effect on SOCE, after 7 days, however. SOCE decreased by 55% (0.2367 ± 0.0283 in control vs. 0.1055 ± 0.0255 in Li^+^ treated cells, *n* = 60, *p* < 0.05) (**Figure 6B**). The amplitude of [Ca^2+^]_i_ transient associated with SOCE decreased by ∼64% (0.2479 ± 0.0205 in control vs. 0.0899 ± 0.0188 in VPA treated cells, *n* = 60, *p* < 0.05) after 3 days and by ∼57% (0.2367 ± 0.0283 in control vs. 0.1008 ± 0.0263 in VPA treated cells, *n* = 60, *p* < 0.05) after 7 days of treatment with 1 mM VPA (**Figure 6C**). In both control and drug-treated cells, [Ca^2+^]_i_ elevation induced by the SERCA inhibitor thapsigargin at 1 μM was not detectable.

## Discussion

A large body of evidence indicates that the resting and stimulated levels of [Ca^2+^]_i_ are elevated in BD (Wasserman et al., 2004 and references therein). It has been noted also that several mood stabilizers affect intracellular Ca^2+^ dynamics in various neuronal cells. For example, CBZ inhibits NMDA-evoked Ca^2+^ influx in rat cerebellar granule cells (Hough et al., 1996), while long-term Li^+^ treatment attenuates NMDA receptor-mediated Ca^2+^ entry in primary cultures of rat cerebellar granule cells (Nonaka et al., 1998) and glutamate-mediated Ca^2+^ signaling in hippocampal neurones (Sourial-Bassillious et al., 2009). Significant reduction of the low-threshold Ca^2+^ current has been observed in rat nodose neurones treated with valproic acid (Kelly et al., 1990). Our present findings suggest that down-regulation of TRPC1 gene expression may be one of the mechanisms of drug action on intracellular Ca^2+^ dynamics in neurones.

Firstly, we found that chronic treatment with all three anti-bipolar drugs, CBZ, Li^+^ and VPA, down-regulated mRNA and protein expression of TRPC1 in a concentration-dependent manner. CBZ at lower concentration (25 μM) decreased TRPC1 gene expression after 7 days of treatment but had no effect after 3 days of treatment. However, both Li^+^ and VPA at lower concentrations (0.5 mM and 100 μM) had no effect in the time period used in the present study. This effect of drugs seems to be independent from the type of neurones. In the present work, we have observed the decrease of TRPC1 expression in cultured cerebellar granule cells, cultured cerebral cortical neurones and freshly isolated neurones from the brains *in vivo*. Previously, we reported similar drug effect in astrocytes (Yan et al., 2013). Secondly, we found a substantial decrease in the SOCE in cerebellar granule cells exposed to each of the three drugs. TRPC1 channels control several aspects of cytosolic ion signaling due to their polyionic permeability, as in physiological conditions TRPC1 channels provide both Ca^2+^ and Na^+^ influx (Reyes et al., 2013). In astrocytes, the TRPC1 protein has been implicated in exocytotic release of glutamate (Parpura et al., 2011).

It has been suggested that TRPC1 are positioned downstream of glutamate metabotropic receptors (mGluRs) and NMDA receptors in neurones. It is of interest because overactivity of glutamatergic system in BD has been documented (Chen et al., 2010), and regulation of TRPC1 function by glutamate receptors may contribute to the pathophysiology of the disease. It has been reported that TRPC1 channels localize in perisynaptic regions of the cerebellar parallel fiber-Purkinje cell synapse where they are physically and functionally coupled with the mGluR1 to induce a slow excitatory post-synaptic conductance (EPSC) (Kim et al., 2003). Knocking down TRPC1 using TRPC1-shRNA or blocking TRPC1 channels reduced glutamate-induced and mGluR5-mediated cell death in murine hippocampal cell line HT22 (Narayanan et al., 2014). The large depolarizing plateau potential which is essential for the epileptiform burst firing by metabotropic glutamate receptors was reduced by 74% in septal neurones of TRPC1^-/-^ mice compared to WT mice (Phelan et al., 2012). In olfactory bulb granule cells TRPC1, as well as TRPC4, can be activated downstream of NMDA receptors and contribute to slow dendritic GABA release (Stroh et al., 2012). These findings suggest that TRPC1 contributes to multiple signals instigated by activation of glutamate receptors, and a decrease of TRPC1 expression and function by anti-bipolar drugs may overcome of the abnormal overactivity of glutamate receptors that develops in BD.

## Conclusion

We demonstrated, for the first time, that gene expression of neuronal TRPC1 is decreased by all three anti-bipolar drugs. Keeping in mind the similar effect in astrocytes, we may conceive that TRPC1 may be a new therapeutic target for BD. It seems that the decrease of TRPC1 gene expression and function affects multiple neurotransmitters in neuronal cells. However, the final conclusion of the pathophysiological relevance depends on further understanding of TRPC1 function in neurones.

## Ethics Statement

All experiments were carried out in accordance with the USA National Institute of Health Guide for the Care and Use of Laboratory Animals (NIH Publications No. 80-23) revised 1978, and all experimental protocols were approved by the Institutional Animal Care and Use Committee of China Medical University.

## Author Contributions

LP conceived and designed experiments; TD, YR, and RF collected and analyzed the data; LP and AV interpreted the data and wrote the paper. All authors commented on the manuscript and have approved the final version.

## Conflict of Interest Statement

The authors declare that the research was conducted in the absence of any commercial or financial relationships that could be construed as a potential conflict of interest.

## Supplementary Material

FIGURE S1 **Original blots of Figure 1B**.

FIGURE S2 **Nifedipine, a blocker of L-type calcium channels did not affect SOCE administered either 1 min after addition of Ca^2+^**(A)** or 2 min before addition of TG (B)**.
