# Supplementary figures and images for: Chronic Treatment with Anti-bipolar Drugs Down-Regulates Gene Expression of TRPC1 in Neurones
Source: Front Cell Neurosci. 2017 Jan 10;10:305. doi: 10.3389/fncel.2016.00305 (PMC5223735; doi:10.3389/fncel.2016.00305)

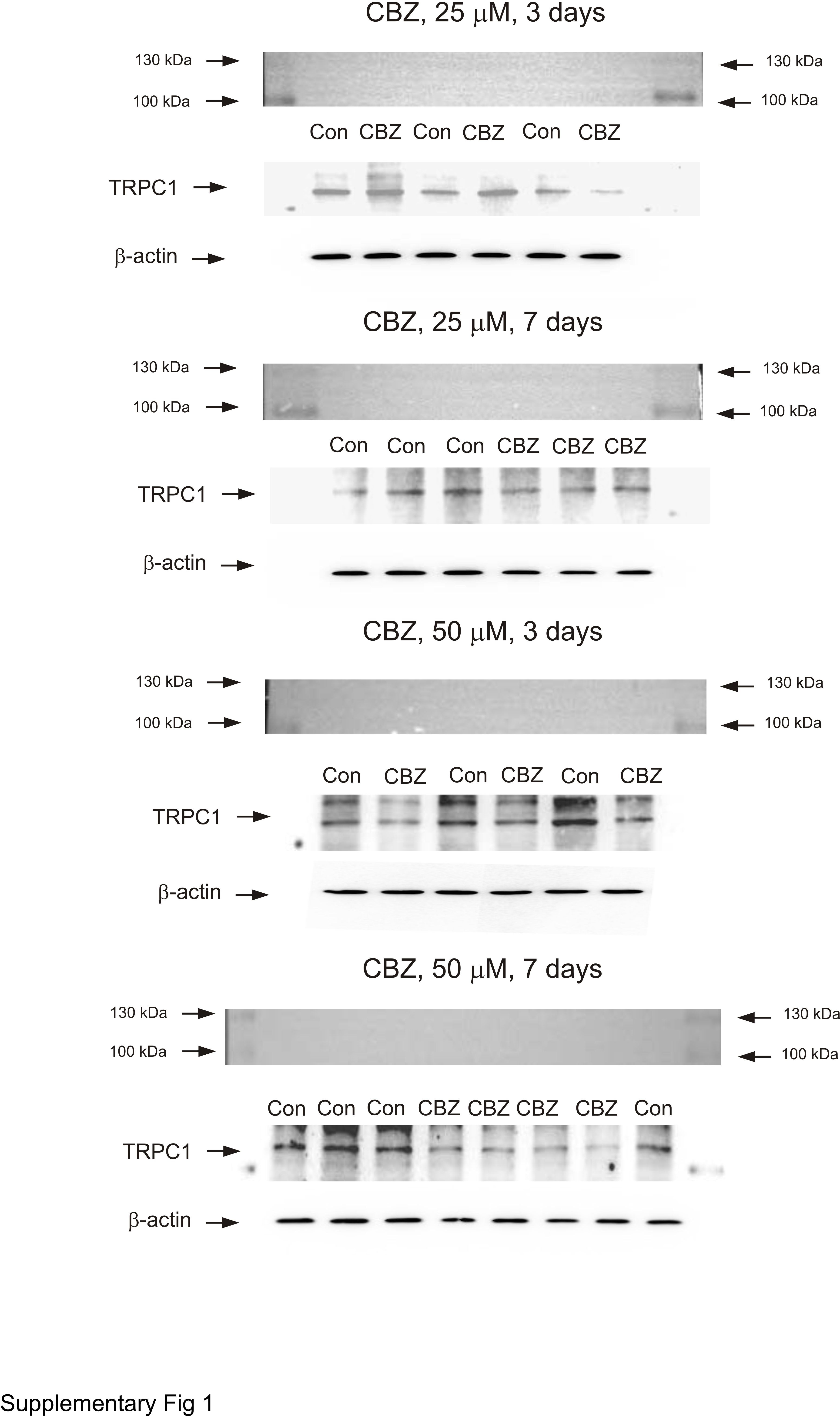

Supplement: FIGURE S1 — Original blots of Figure 1B. [file Image_1.TIF]

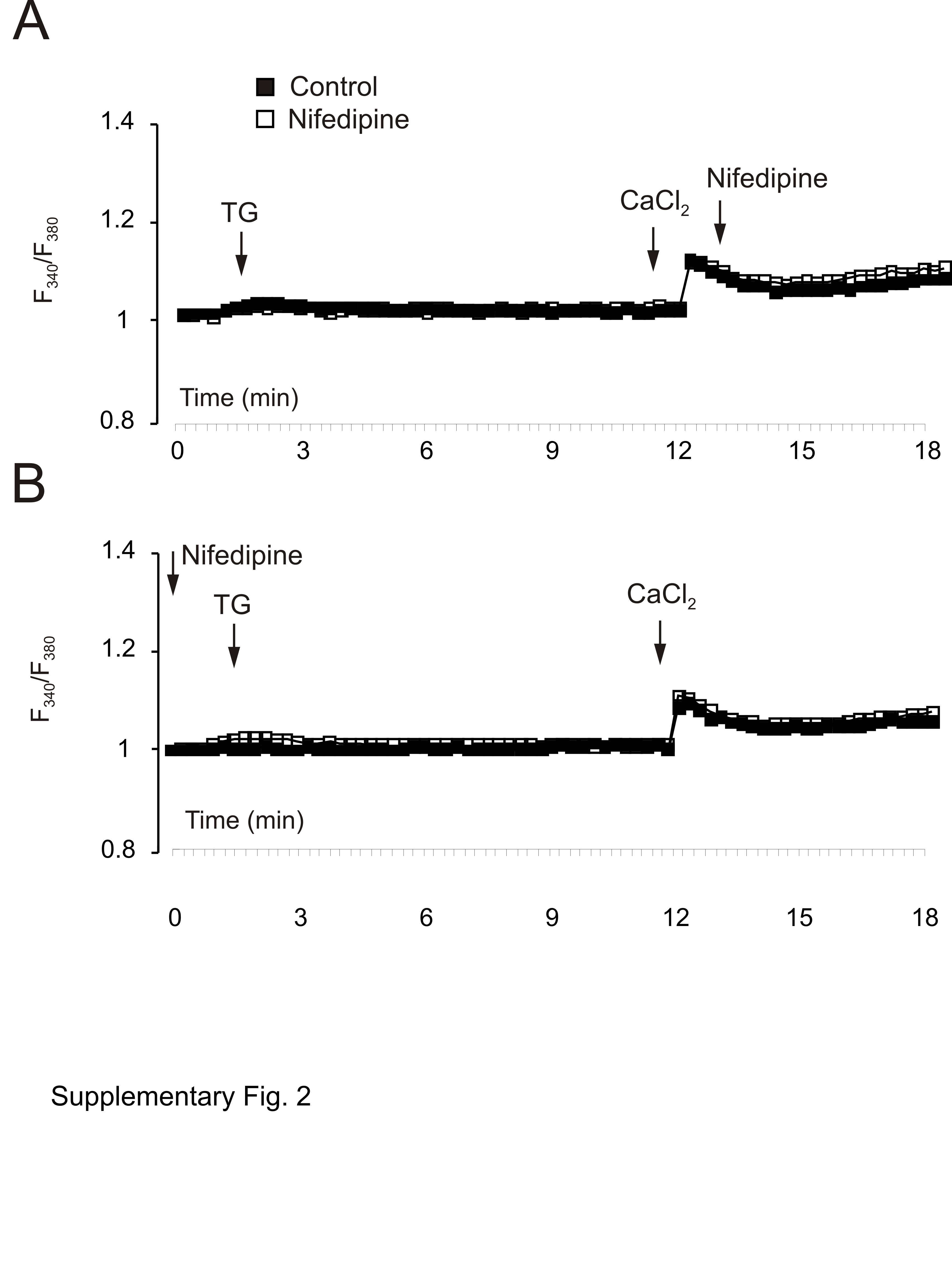

Supplement: FIGURE S2 — Nifedipine, a blocker of L-type calcium channels did not affect SOCE administered either 1 min after addition of Ca2+(A) or 2 min before addition of TG (B). [file Image_2.TIF]
